# New Paradigms to Assess Consequences of Long-Term, Low-Dose Curcumin Exposure in Lung Cancer Cells

**DOI:** 10.3390/molecules25020366

**Published:** 2020-01-16

**Authors:** Gintare Smagurauskaite, Jagdish Mahale, Karen Brown, Anne L. Thomas, Lynne M. Howells

**Affiliations:** Leicester Cancer Research Centre, University of Leicester, Robert Kilpatrick Clinical Sciences Building, Leicester Royal Infirmary, Leicester LE2 7LX, UK; g.smagu@yahoo.com (G.S.); jagdish.mahale@kennedy.ox.ac.uk (J.M.); kb20@le.ac.uk (K.B.); at107@le.ac.uk (A.L.T.)

**Keywords:** curcumin, lung cancer, resistance, chemotherapy

## Abstract

Curcumin has been investigated extensively for cancer prevention, but it has been proposed that long-term treatments may promote clonal evolution and gain of cellular resistance, potentially rendering cancer cells less sensitive to future therapeutic interventions. Here, we used long-term, low-dose treatments to determine the potential for adverse effects in non-small cell lung cancer (NSCLC) cells. IC_50_s for curcumin, cisplatin, and pemetrexed in A549, PC9, and PC9ER NSCLC cells were evaluated using growth curves. IC_50_s were subsequently re-assessed following long-term, low-dose curcumin treatment and a three-month treatment withdrawal period, with a concurrent assessment of oncology-related protein expression. Doublet cisplatin/pemetrexed-resistant cell lines were created and the IC_50_ for curcumin was determined. Organotypic NSCLC-fibroblast co-culture models were used to assess the effects of curcumin on invasive capacity. Following long-term treatment/treatment withdrawal, there was no significant change in IC_50_s for the chemotherapy drugs, with chemotherapy-resistant cell lines exhibiting similar sensitivity to curcumin as their non-resistant counterparts. Curcumin (0.25–0.5 µM) was able to inhibit the invasion of both native and chemo-resistant NSCLC cells in the organotypic co-culture model. In summary, long-term curcumin treatment in models of NSCLC neither resulted in the acquisition of pro-carcinogenic phenotypes nor caused resistance to chemotherapy agents.

## 1. Introduction

Lung cancer is the most commonly diagnosed cancer worldwide, and is the leading cause of cancer mortality [1]. Despite many treatment advances, one-year survival for those with stage IV disease remains at less than 20% [2], emphasising that a focus on early detection and prevention is essential for more effectively managing disease burden. Whilst lung cancer prevention is primarily focused around smoking cessation, there may still be a role for therapeutic prevention (chemoprevention) approaches in certain high-risk cohorts. 

Historically, therapeutic prevention strategies have had little success in lung cancer, with two large trials (alpha-tocopherol and beta-carotene (ATBC), beta-carotene and retinol (CARET)) showing increased mortality in smokers following beta-carotene intervention [3,4]. Subsequent investigations suggested that beta-carotene and its metabolites may contribute to neutrophil-induced genotoxicity in smokers [5], with smoking being a risk factor in itself, independent from inhaled levels of tar and nicotine [6]. These study failures have provided critical information and have led to rational changes to the way in which prevention trials are now undertaken. Chemoprevention trials are often in healthy populations and so should give considerable scrutiny to factors that would decrease the likelihood of adverse outcomes, including: choice of dose, when to treat, dosing strategy, in-depth knowledge of molecular mechanisms and recognition/planning for previously unknown toxic mechanisms, identification of which populations may benefit/be at risk, and choice of most suitable efficacy biomarkers for early read-out [7]. 

A further safety consideration for chemoprevention trials is to understand the potential effects that long-term dosing may have at a cellular level in those individuals that subsequently go on to develop cancer. It has been suggested that long-term single-agent use in this paradigm may result in the promotion of clonal evolution and generation of therapy-resistant subclones [8], potentially resulting in decreased efficacy of standard-of-care chemotherapy. Gain of resistance to anti-cancer therapeutics and small molecule inhibitors is a common occurrence even when administered over relatively short time frames. Within the chemoprevention setting, preventive drugs are typically administered over longer time frames meaning that gain of resistance is also common (e.g., selective oestrogen receptor modulators in breast cancer prevention [9,10]). It is therefore imperative to better understand the consequences of longer-term dosing using preventive agents, to determine whether there may be adverse implications for the effectiveness of future therapies. 

The diet-derived agent, curcumin, has been investigated extensively for use as a cancer preventive agent, but the issue around potential for gain of resistance following long-term administration, which may be implicated in some of the notable failures of intervention approaches based around administration of dietary constituents, has yet to be investigated [8]. Using multiple experimental approaches, we tried to address these concerns. We firstly attempted identification of models potentially useful in the lung cancer chemoprevention setting [11], and subsequently generated and applied these models using curcumin as an exemplary agent. We then assessed the effects of long-term, low-dose curcumin intervention and its subsequent withdrawal on lung cancer cell lines. Furthermore, cell lines resistant to combinations of commonly used NSCLC chemotherapy drugs were developed to ascertain whether chemotherapy-resistant cell lines retained sensitivity to curcumin. Finally, we developed a 3D air-interface organotypic co-culture model of lung cancer to assess the effects of curcumin on invasive capacity.

## 2. Results

### 2.1. Effect of Long-Term Curcumin Treatment and Its Withdrawal on Cellular Sensitivity to Chemotherapy Agents

Sensitivity to single-agent cisplatin and pemetrexed in native cell lines, cells treated for 3 months with 0.25 μM of curcumin, and cells that had a subsequent three-month withdrawal of curcumin are shown in Table 1 and Supplemental Figure S1A,B. No significant differences in any of the cell lines’ response to cisplatin or pemetrexed following long-term treatment with curcumin or its subsequent withdrawal were observed.

### 2.2. Sensitivity of Chemotherapy-Resistant Cell Lines to Curcumin

We then sought to determine whether chemotherapy-resistant cell lines still exhibited sensitivity to curcumin. IC_50_ values in native cell lines at 120 h and 168 h following curcumin treatment revealed the order of sensitivity to curcumin to be PC9ER > PC9 > A549. This order of sensitivity was maintained following the generation of resistance, and IC_50_s did not significantly differ between native and chemotherapy-resistant cell lines (Table 2, Supplemental Figure S2) showing that gain of resistance to chemotherapy agents did not alter sensitivity to curcumin.

### 2.3. Effect of Long-Term Curcumin Treatment and Its Withdrawal on Expression of Oncology Array Proteins

Oncology arrays were used to ascertain whether long-term curcumin treatment adversely affected the expression of oncology-related proteins in the lung cancer cell lines, and whether any changes were reversed upon treatment withdrawal. Significant changes in protein expression for each cell line following long-term curcumin treatment and its withdrawal are shown in Table 3 (Supplemental Figures S3–S8). A549 cells were the most resistant to significant changes induced by curcumin, and PC9ER cells were the most sensitive. The majority of changes saw the downregulation of proteins, with most of this downregulation affecting pro-carcinogenic proteins (73% were pro-carcinogenic across all three cell lines).

Of note, the pro-carcinogenic proteins downregulated following long-term curcumin treatment and its withdrawals were associated with poor prognosis, progression, invasion, and epithelial-mesenchymal transition in lung cancer.

### 2.4. Effect of Curcumin on Invasive Capacity of Lung Cancer Cells Using the Organotypic Co-Culture Model

**Figure 1:**
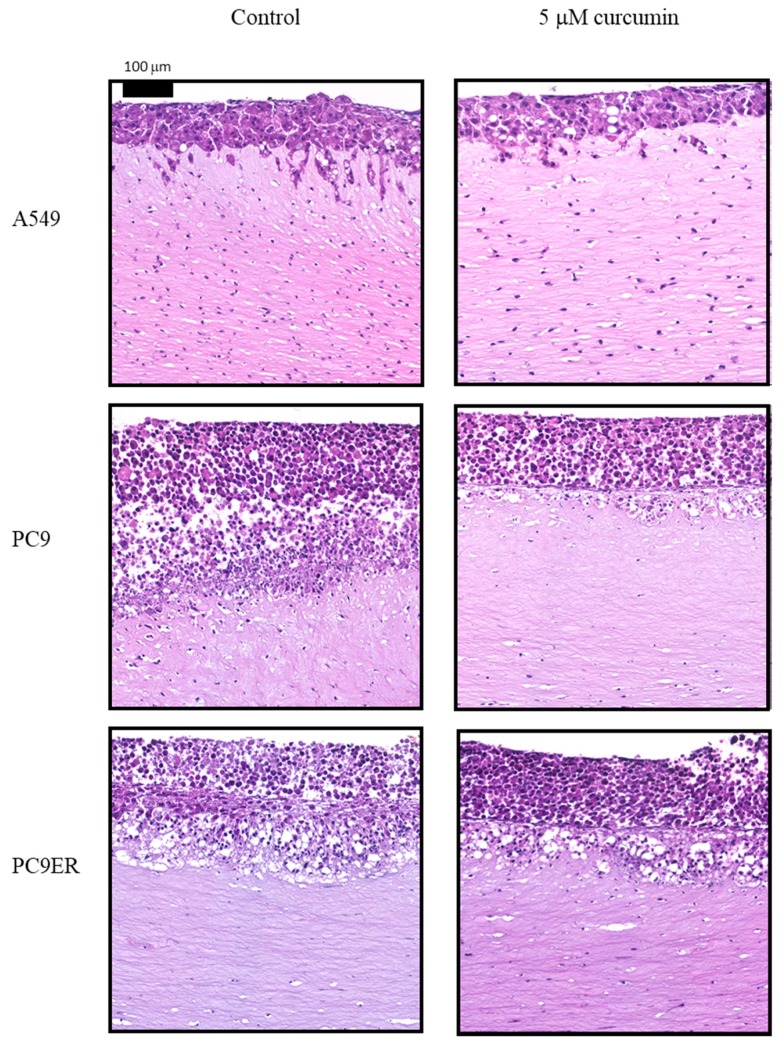
Representative haematoxylin and eosin (H&E) images (×20 magnification) of the organotypic co-culture model for A549, PC9, and PC9ER native cell lines following treatment with 5 μM of curcumin. Co-cultures were performed on three separate occasions for each cell line.

The invasive capacity of native lung cancer cell lines and their doublet-resistant counterparts in response to curcumin was assessed using the organotypic co-culture model (Table 4, Figure 1). Curcumin treatment significantly reduced invasion at 0.5 μM for A549, PC9, and PC9ER lung cancer cell lines. In the resistant cell lines, A549^cisR/pemR^ exhibited decreased invasion at 5 μM only, whereas invasion by PC9^cisR/pemR^ and PC9ER^cisR/pemR^ was significantly reduced at both 0.5 and 5 μM by up to 56%. Long-term, low-dose curcumin treatment and treatment withdrawal did not significantly alter invasive capacity when compared to native cell lines (data not shown).

### 2.5. Effect of Curcumin on MRC5 Fibroblasts

**Figure 2:**
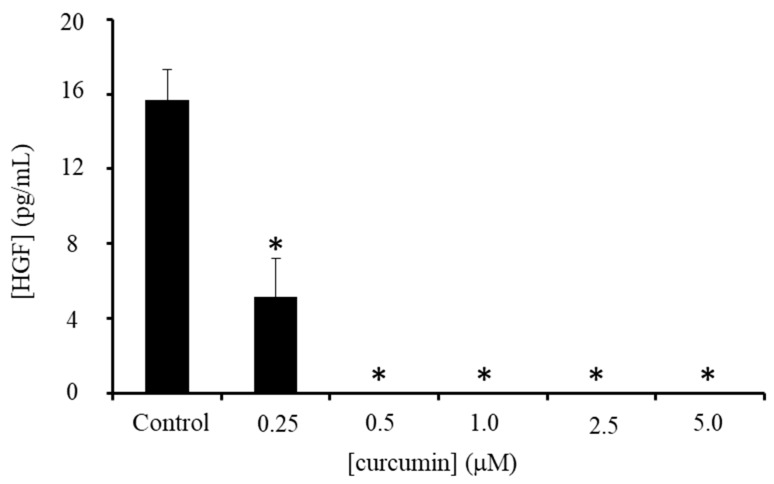
MRC5-secreted hepatocyte growth factor (HGF) protein levels in media following a seven-day treatment with curcumin. Bars represent mean of three independent experiments ±SD, * *p* ≤ 0.05.

Neither the native nor resistant lung cancer cell lines had the capacity to invade without the presence of MRC5 fibroblasts in the co-culture model. The c-Met (mesenchymal–epithelial transition) pathway is a key regulator of migratory phenotype, with the c-Met ligand, hepatocyte growth factor (HGF) secreted in high levels by MRC5 cells. MRC5 cells themselves were sensitive to curcumin, with an IC_50_ of 1.49 ± 0.3 μM at 144 h (Supplemental Figure S9), and so the decrease in invasion following curcumin treatment may have been in part due to a decrease in MRC5 cell number. However, H&E staining of the co-cultures reveals in-gel fibroblasts to still be numerous, suggesting that curcumin may also be directly affecting signalling networks between cancer and stromal cells. Following curcumin treatment, HGF secretion by fibroblasts (normalised to cell number to take into account any decreases) was decreased by 70% at 0.25 μM and completely abrogated at higher doses (Figure 2). 

### 2.6. Effect of MRC5 HGF Knockdown on Invasive Capacity of Tumour Cells in the Organotypic Co-Culture Model

**Figure 3:**
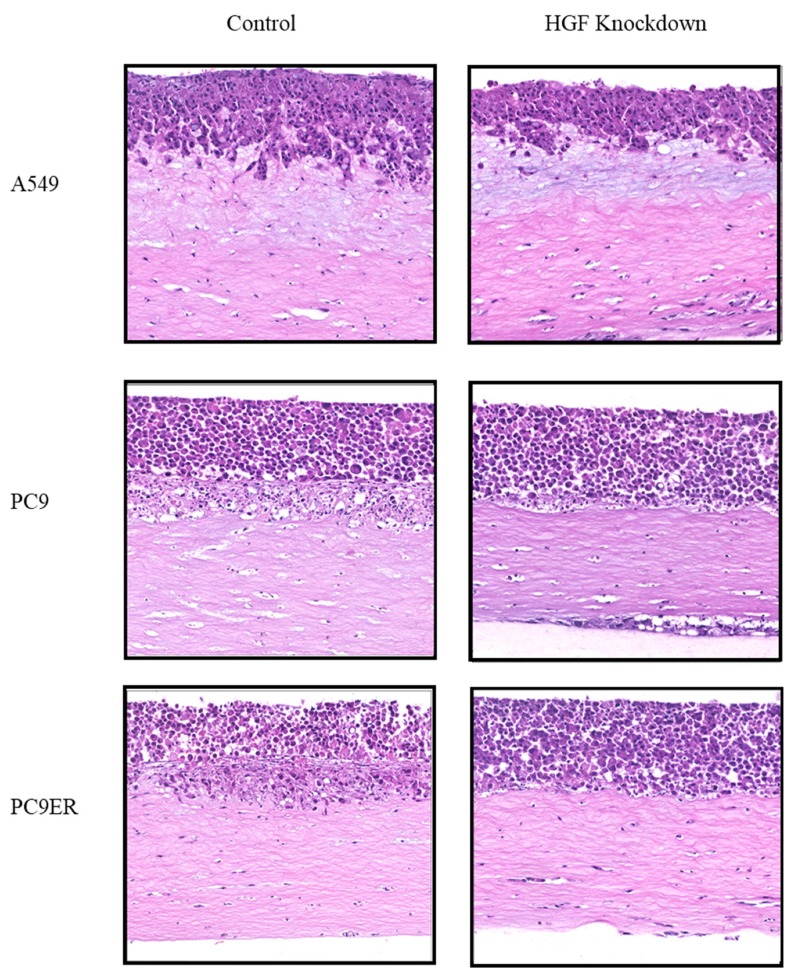
Representative H&E images (×20 magnification) showing effects of HGF knockdown in MRC5 cells on the invasive front of lung cancer cell lines in organotypic co-culture compared to their native cell counterparts. Co-cultures were performed on three separate occasions.

In order to ascertain whether the abrogation of fibroblast-secreted HGF by curcumin was responsible for decreased invasive capacity of tumour cells in the co-culture model, we used lentiviral transduction to create a stable MRC5 HGF knockdown. Following knockdown, HGF concentrations decreased from 14.15 pg/mL/5000 cells to 0.81 pg/mL/5000 cells, constituting a 94% decrease in HGF production (Supplemental Figure S10). Once the success of this approach had been established, the HGF knockdown MRC5 cells (MRC5^-HGF^) were co-cultured with the lung cancer cell lines (Figure 3). Co-culture with MRC5^-HGF^ cells resulted in decreased invasive capacity for all cell lines, reaching significance for PC9 and PC9ER (Table 5), with decreases in invasion of 53% and 58%, respectively.

## 3. Discussion

Understanding the consequences and mechanisms of therapeutic resistance is of vital importance, but to date has been given little consideration in preventive medicine that uses low doses of well-tolerated drugs over many years. We have previously highlighted the importance of improving the variety and range of models that should be made available to investigate primary, secondary, and tertiary cancer chemoprevention strategies [11], and this article presents the first tranche of these approaches.

Curcumin is known to mitigate pro-carcinogenic signalling across numerous pathways. Here, we evaluated whether long-term exposure to low-dose curcumin affected the response of cells to the chemotherapy agents, cisplatin and pemetrexed, used as first-line chemotherapy in NSCLC of non-squamous histology with no known *EGFR* sensitising mutation, and also at relapse for those with *EGFR* mutation. Several reports have previously suggested that curcumin may offer a sensitising effect when administered at high doses in conjunction with chemotherapy drugs [12,13,14,15]. When given at low-dosage over three months (which is more consistent with administration in a preventive setting), no sensitising effect was observed in three lung cancer cell lines exhibiting different mutational spectra (A549: *K-ras* mutant; PC9: *EGFR* driver mutation; PC9ER: *EGFR* driver mutation but exhibiting erlotinib resistance). Importantly, there was no evidence of decreased sensitivity to chemotherapy drugs following curcumin treatment and its subsequent withdrawal, suggesting that long-term, low-dose curcumin is insufficient to drive clonal evolution and promote therapeutic resistance. This is an important consideration when planning any long-term therapy regimen that may have the potential to impact on the success of future treatments. 

These findings were corroborated by the oncology array platform, which demonstrated that long-term, low-dose curcumin favours upregulation of anti-carcinogenic, and downregulation of pro-carcinogenic proteins, many of which are associated with epithelial to mesenchymal transition (EMT). Curcumin has previously been shown to play a role in preventing or reversing the generation of an EMT phenotype [16,17,18,19], albeit at single, non-pharmacologic high doses ranging from 10 to 80 μM. Such high doses are unlikely to be attained clinically, making it difficult to interpret and translate mechanistic relevance to prevention strategies that are typified by sub-micromolar systemic curcuminoid availability. High-dose curcumin has been shown specifically to inhibit the EMT-associated proteins snail [20], lumican [21], CCL2 [22], cathepsin B [23], carbonic anhydrase [24], and matrix metalloproteinase-3 [25], all of which we also show here to have been significantly downregulated following long-term, low-dose curcumin. Some of these proteins exhibited prolonged downregulation even following a three-month withdrawal period from curcumin treatment. This alludes to the ability of curcumin to decrease the invasive potential of NSCLC cells in vitro following prolonged dosing at a concentration for which pharmacologic benefit is not usually observed in cellular models following short-term treatments.

We next sought to create models representative of an acquired resistant phenotype in lung cancer. Despite NSCLC rarely being treated by monotherapy alone, the A549/DDP cell line has been used extensively as a model of acquired cisplatin chemoresistance. To expand the relevance of this model, we generated lung cancer cell lines resistant to cisplatin and pemetrexed doublet therapy, and together with the PC9ER erlotinib-resistant cell line, further generated a model with a triplet resistant phenotype. All resistant cell lines were similarly as sensitive to curcumin as their native counterparts, which contrasts with previous studies using the A549/DDP cells that exhibited decreased sensitivity [26]. Following the generation of doublet/triplet resistance, cells took on distinct spindle-like morphological characteristics, suggestive of EMT phenotypical gain. This was further investigated via the oncology array platform, which revealed significant upregulation of vimentin across all three cell lines (Smagurauskaite et al., unpublished data).

Due to the ability of curcumin to downregulate the expression of pro-EMT proteins in all cell lines, we next sought to apply this knowledge to an air-interface model of invasion. In the organotypic co-culture model, lung cancer cells are not directly submerged in treatment-containing media, but instead, treatments pass through a basement membrane/collagen matrix prior to reaching the target cancer cells. This model has the advantage that it allows the addition of other cell types to enable specific cell–cell interactions to be further elucidated. Despite expression of EMT-associated proteins, neither the native cell lines nor their resistant counterparts were able to invade through collagen gels when in monoculture, and required co-culture with MRC5 fibroblasts to facilitate their invasion. 

The role of fibroblasts in creating a permissive and motility-inducing microenvironment in lung cancer has been well established (reviewed in [27]), together with their ability to facilitate the acquisition of therapeutic resistance [28]. Fibroblasts are a rich source of many growth factors including hepatocyte growth factor (HGF), which has been shown to be a key factor in enhancing NSCLC progression [29]. Paracrine activation of the Met (HGF) receptor in NSCLC occurs upon binding of the active HGF molecule. Met is a receptor tyrosine kinase regulated by phosphorylation at a number of sites: pMet1003 regulates ubiquitination and degradation of the receptor; pMet1234 and pMet1235 in the tyrosine kinase domain regulate kinase activity; pMet1349 and pMet1356 in the c-terminal domain serve as docking sites for downstream proteins [30]. Phosphorylation of sites within the tyrosine kinase domain following HGF binding results in receptor dimerisation and activation, leading to the consequent activation of a number of pro-carcinogenic downstream signalling pathways including those involved in EMT, invasion, angiogenesis, and proliferation. We hypothesised that the ability of curcumin to downregulate HGF production from fibroblasts in the co-culture model may be responsible for the reduced invasive capacity of NSCLC cells, rather than just a direct effect of curcumin on the cancer cells per se. The importance of curcumin’s ability to decrease HGF secretion by the MRC5 cells was confirmed following the creation of MRC5^-HGF^ cells and their addition to the organotypic model, resulting in significantly decreased tumour cell invasion. Conversely, Jiao et al. [17] confirmed that the HGF-induced mesenchymal morphology of A549 and PC9 cells could be abrogated when the cancer cells themselves were directly treated with 10–30 μM of curcumin, in addition to downregulating HGF-induced downstream PI3K signalling. This leads to the notion that curcumin is able to decrease Met signalling via numerous different mechanisms, some of which are summarised in Supplementary Figure S11 (adapted from [31]). 

There is increasing emphasis on the importance of the c-Met/HGF pathway in lung cancer progression and promotion of resistance resulting from Met amplification, exon 14 mutation, or activation due to HGF expression [32,33,34], with small molecule Met inhibitors and anti-HGF antibodies now in clinical trials [35,36]. Exploring further mechanisms by which curcumin may inhibit HGF signalling in lung cancer should contribute to the mechanistic scrutiny applied when considering the utility of curcumin in therapeutic prevention regimens.

## 4. Methods

### 4.1. Cell Lines

Non-small cell lung cancer (NSCLC) accounts for approximately 87% of lung cancer cases and can be subdivided into adenocarcinoma, squamous cell carcinoma, and large cell carcinoma. Adenocarcinomas are the most common, with treatment regimens including cisplatin/pemetrexed/erlotinib [37]. Adenocarcinoma cell lines with representative common driver mutations A549 (*K-ras* mutant), PC9 (*EGFR* erlotinib-sensitive mutant), its erlotinib-resistant derivative cell line PC9ER, and MRC5 fibroblasts were originally obtained from the American Type Culture Collection (ATCC). All three NSCLC cell lines were cultured in RPMI-1640 (ThermoFisher, Leicester, UK) and MRC5 were cultured in DMEM-6429 (ThermoFisher, Leicester, UK), supplemented with 10% FCS and l-Glutamine. Cell lines were regularly tested for mycoplasma contamination. 

### 4.2. Assessing Cell Proliferation in Response to Curcumin, Pemetrexed, and Cisplatin

A549 cells (1000/well), PC9, and PC9ER cells (2000/well) were seeded in 24-well plates and allowed to adhere overnight prior to treatment. Cells were treated with increasing concentrations of curcumin (0.25–10 μM), pemetrexed (0.05–1.0 μM), or cisplatin (0.05–10 μM). Stocks of curcumin were reconstituted in DMSO, cisplatin in PBS/140 mM NaCl, and pemetrexed disodium in PBS. All curcumin treatments contained equivocal amounts of DMSO, which did not exceed 0.05%. Cells were counted at 72 h, 96 h, 120 h, 144 h, and 168 h time points using a Beckman Coulter Z2 particle analyser (Beckman Coulter, High Wycombe, UK). Cell counts were subsequently plotted to determine the concentrations at which cellular proliferation was reduced by 50% (IC_50_). Curcuminoid concentrations up to 18.85 μg/mg (51 μM) have previously been observed in human colorectal biopsy material (even after bowel preparation and extensive sample wash-out) following 2.35 g daily oral curcumin consumption [38]. In addition, studies using the Meriva curcumin formulation in human healthy volunteers revealed the total plasma curcuminoid C_max_ to be 206.9 ng/mL (equating to ~0.56 μM of curcuminoids) following a dose of 396 mg curcuminoids contained in the Meriva formulation [39]. Hence, for long-term treatments, we deemed 0.25 μM to be a low in vitro dose, commensurate with clinically achievable concentrations available systemically.

### 4.3. Long-Term Treatments with Curcumin

Cell line treatments using curcumin are typically for 1 week or less, using cytotoxic concentrations of up 50 μM. In order to better model a long-term treatment, we sought to extend treatment times 12-fold out to 3 months. Whilst this is not directly comparable to clinical dosing schedules for preventive agents, this time frame equates to approximately 20 cell passages and thus minimises clonal instability as a consequence of excessive passaging. A549, PC9, and PC9ER cells were cultured in RPMI-1640 media supplemented with 0.25 µM of curcumin (Indena S.p.A.). Following 3 months of growth in curcumin-supplemented media, cells were used for sensitivity analyses. Cells were subsequently passaged for a further 3 months in curcumin-free media to assess effects of curcumin withdrawal 

### 4.4. Generation of Cell Lines Resistant to Both Cisplatin and Pemetrexed

All NSCLC cell lines were cultured in media containing increasing concentrations of pemetrexed disodium (Santa Cruz Biotechnology, Heidelberg, Germany) together with cisplatin (cis-Diammineplatinum(II) dichloride, Sigma, Poole, UK) to achieve cell lines exhibiting resistance to drug concentrations approximately 10 × IC_50_ values. For A549 cells, resistance was achieved when cells were able to grow in media containing 10 µM cisplatin/0.6 µM pemetrexed; for PC9 cells, 2 µM cisplatin/0.1 µM pemetrexed; for PC9ER cells, 6.7 µM cisplatin/0.1 µM pemetrexed. Double-resistant cell lines were designated A549^cisR/pemR^, PC9^cisR/pemR^, and PC9ER^cisR/pemR^.

### 4.5. Oncology Arrays

Proteome Profiler Human XL Oncology Array Kits (R&D Systems, Abingdon, UK) contain 84 human cancer-related proteins spotted in duplicate onto a nitrocellulose membrane. The array kits allowed comparison between native and resistant cell lines or those that had undergone long-term, low-dose curcumin treatment in order to determine whether significant changes to oncological profiles had occurred. Lysates were first prepared for all of the relevant cell lines using lysis buffer supplied with the kit. Oncology array membranes were blocked in 2 mL of Array Buffer 6 for 1 h prior to adding samples containing 200 µg of protein for incubation overnight at 4 °C. Arrays were washed ×3 with oncology array wash buffer and incubated with Detection Antibody Cocktail for 1 h at room temperature. Following a further three washes, arrays were incubated with 2 mL of 1 × Streptavidin-HRP (R&D Systems, Abingdon, UK) for 30 min at room temperature. Following a further three washes, the signal was developed with Chemi Reagent Mix for 1 min. Data analysis was performed by measuring the intensity of each spot using GeneSys software v1.5.4.0 (GeneSys, Camberly, UK) and comparing mean values between control and treated array membranes.

### 4.6. Organotypic Co-Culture

Co-cultures are useful models of invasive capacity which take into consideration effects of components of the tumour microenvironment. Co-cultures were carried out in a similar manner to that described previously [40], but with differing cell lines as below:

In brief, collagen gels were made by plating 1 mL of total volume, containing 350 µL (3.5 parts) of rat-tail collagen I (Millipore, Watford, UK), 350 µL (3.5 parts) of Matrigel (Corning, High Wycombe, UK), 100 µL (1 part) of 10 × DMEM, 100 µL (1 part) of sterile-filtered FCS, and 100 µL (1 part) of MRC5 cell suspension (2.5 × 10^6^ cells/mL) for gels with fibroblasts or 100 μL of 10% DMEM for gels that did not contain fibroblasts. Gels were set for 1 h, then 1 mL of 10% DMEM was added, and the gels were incubated at 37 °C, 5% CO_2_ overnight. The following day, cell suspensions of 250,000 NSCLC cells/mL and 1.25 × 10^6^ MRC5/mL were prepared and mixed to give a 1:5 ratio of NSCLC:MRC5 cells. One millilitre of the cell mixture was added drop-wise to the collagen gels, which were subsequently incubated overnight at 37 °C, 5% CO_2_. All other aspects were as previously described in [40]. For the co-culture of resistant cell lines, cell numbers had to be re-optimised with cell ratios of 1:5 for A549^cisR/pemR^:MRC5, 1:2 PC9^cisR/pemR^:MRC5, and 5:1 PC9ER^cisR/pemR^:MRC5.

After 12 days of supplementing the co-cultures with media containing 0.5–5 μM of curcumin, gels were formalin-fixed and paraffin-embedded. Cut sections were stained with haematoxylin and eosin (performed as a service by the University of Leicester Core Biotechnology Services Histopathology Laboratory, as per standard diagnostic protocols) and analysed using a Hamamatsu NanoZoomer Digital Slide Scanner (Hamamatsu, Welwyn Garden City, UK) to visualise cell invasion. Invasion was subsequently quantified using ImageJ (version 1.49, open source software) using the following formula: Percent invasion = total invaded area/total area × 100.

### 4.7. HGF ELISA

MRC5 cells were seeded into 24-well plates (1 × 10^3^/well), left to adhere overnight, and the culture medium replaced by fresh media containing 0–5 μM of curcumin. Following 7 days of treatment, media was removed and the remaining cells were counted to determine HGF levels/5000 cells using a human HGF ELISA kit (ThermoFisher, Leicester, UK) following the manufacturer’s instructions. 

### 4.8. Generation of Stable HGF Knockdown in MRC5 Cells

Four 29mer plasmids containing Hu HGF shRNA in pGFP-shLenti vector constructs and one scrambled negative control (see supplementary information for sequences) (OriGene, Rockville, MD, USA) were used to transform library efficiency DH5a competent cells, with plasmid DNA extracted using a Qiagen Plasmid Maxiprep kit as per manufacturer’s instructions (Qiagen, Crawley, UK).

To generate lentiviral particles, 5 μg of the pLenti-shRNA construct was combined with 6 mg of packaging plasmids and 500 μL Opti-MEM (ThermoFisher, Leicester, UK). Following the addition of MegaTran transfection reagent (OriGene, MD, USA), the mixture was added to 70% confluent HEK293T cells for 12 h, after which the media was changed. Following overnight incubation, viral particle-containing supernatant was harvested, fresh media added for a further overnight incubation, and the second batch of media collected and combined with the first to give a viral titre of between 10^6^ and 10^7^ TU/mL. To perform the stable HGF knockdown in MRC5 cells, MRC5 cells were seeded at 1 × 10^5^ on to 12-well plates and allowed to adhere overnight. The desired number of viral particles (multiplicity of infection (MOI) ranging from 20 to 150) were added to the cells and incubated for 18 h before replacing with fresh media overnight followed by splitting at a 1:10 ratio. Cells for each MOI were grown in a range of puromycin-containing medium (0–1.5 μM) until resistant subclones could be identified and sub-cultured on. Secreted HGF levels were assessed for all clones using the human HGF ELISA kit.

## 5. Conclusions

The use of low-dose chronically administered curcumin in cellular models lends greater relevance to mechanistic outcomes as it represents a pharmacologically relevant concentration that may be achievable within a clinical setting. The ability of low-dose curcumin to decrease the EMT phenotypes of cancer cells in conjunction with targeting HGF secretion by fibroblasts lends support for potential use in a long-term prevention or maintenance setting, particularly where low toxicity remains a priority.

When administered at low doses over a prolonged period of time, curcumin neither contributed to pro-carcinogenic clonal evolution nor affected the response of NSCLC cells to standard chemotherapeutic intervention, allaying fears over resistance-gain following long-term administration.

## Figures and Tables

**Table 1:** IC_50_s following 168 h treatment with cisplatin and pemetrexed in lung cancer cell lines A549, PC9, and PC9ER. IC_50_s represent those for native cell lines, those that have undergone three-month treatment with 0.25 μM of curcumin, and those with subsequent three-month curcumin withdrawal. Data represent means from three independent growth curve experiments ±SD.

	Treatment	Native Cell Line IC_50_ (±SD)	Long-Term Curcumin Treatment IC_50_ (±SD)	Curcumin Withdrawal IC_50_ (±SD)
**A549**	Cisplatin	1.12 μM (±0.11)	1.20 (±0.43)	1.02 (±0.14)
	Pemetrexed	0.06 μM (±0.01)	0.08 μM (±0.07)	0.08 μM (±0.05)
**PC9**	Cisplatin	0.3 μM (±0.03)	0.42 μM (±0.35)	0.25 μM (±0.18)
	Pemetrexed	11.42 nM (±0.83)	11.42 nM (±1.54)	9.20 nM (±4.26)
**PC9ER**	Cisplatin	0.94 μM (±0.20)	0.57 μM (±0.42)	0.43 μM (±0.34)
	Pemetrexed	10.2 nM (±1.99)	10.20 nM (±2.42)	12.93 nM (±2.89)

**Table 2:** IC_50_s of native and double-resistant lung cancer cell lines in response to curcumin treatments over 168 h. Data represent mean IC_50_ values from three independent growth curve experiments ±SD.

	120 h IC_50_ (±SD)	168 h IC_50_ (±SD)
**A549**	14.67 μM (±1.03)	30.26 μM (±20.87)
**A549^cisR/pemR^**	12.43 μM (±2.43)	15.32 μM (±4.39)
**PC9**	8.55 μM (±5.54)	14.44 μM (±10.52)
**PC9^cisR/pemR^**	9.22 μM (±3.47)	11.15 μM (±4.46)
**PC9ER**	7.33 μM (±1.43)	9.19 μM (±2.14)
**PC9ER^cisR/pemR^**	7.77 μM (±1.41)	8.37 μM (±0.21)

**Table 3:** Effects of a three-month curcumin treatment (0.25 μM) and subsequent three-month curcumin withdrawal on lung cancer cell lines compared to native (untreated) cell lines. Changes in protein expression were assessed using oncology arrays. Arrays were performed in triplicate for A549, PC9, and PC9ER cell lines. Proteins with a known function in lung cancer that were significantly altered (*p* < 0.05) are shown in the table (all data are shown in Supplemental Figures S3–S8). ‘PRO’ represents pro-carcinogenic proteins; ‘ANTI’ represents anti-carcinogenic proteins. Numbers in brackets represent mean percent change compared to native cell lines. Red represents upregulated proteins. Green represents downregulated proteins.

Long-Term Curcumin Treatment					Treatment Withdrawal			
**Cell Line**	Significantly upregulated		Significantly downregulated		Significantly upregulated		Significantly downregulated	
	PRO	ANTI	PRO	ANTI	PRO	ANTI	PRO	ANTI
**A549**	-	-	Kallikrein 6 (26%)	Lumican (38%)	-	-	Vimentin (38%)	-
	-	-	-	Progesterone receptor (32%)	-	-	HO-1 (23%)	-
	-	-	-	Serpin B5/maspin (29%)	-	-	FGF basic (4%)	-
**PC9**	Enolase 2	Serpin B5/Maspin (18%)	VCAM-1 (30%)	Lumican (43%)	-	-	Osteopontin (37%)	Endoglin (26%)
	-	P53 (11%)	CCL2 (29%)	-	-	-	M-CSF (30%)	SPARC (23%)
	-	-	-	-	-	-	Cathepsin B (23%)	Progesterone Receptor (18%)
	-	-	-	-	-	-	VCAM1 (21%)	E-cadherin (14%)
	-	-	-	-	-	-	Progranulin (18%)	-
	-	-	-	-	-	-	IL-2α (18%)	-
	-	-	-	-	-	-	CA125 (16%)	-
	-	-	-	-	-	-	HIF-1 α (14%)	-
	-	-	-	-	-	-	PECAM1 (13%)	-
	-	-	-	-	-	-	Mesothelin (7%)	
**PC9ER**	-	-	Endostatin (51%)	Angiopoetin-1 (38%)	-	-	Amphiregulin (46%)	Angiopoetin-like 4 (44%)
	-	-	eNOS (43%)	FOXO1 (37%)	-	-	Enolase-2 (38%)	Serpin B5/Maspin (30%)
	-	-	α-fetoprotein (42%)	SPARC (28%)	-	-	Cathepsin B (38%)	FOXO1 (16%)
				Lumican (33%)				
	-	-	HCG (41%)	Angiopoetin-like 4 (28%)	-	-	Carbonic Anhydrase IX (30%)	-
	-	-	Cathepsin B (37%)	Prostasin (22%)	-	-	Endostatin (29%)	-
	-	-	ENPP/Autotaxin (37%)	Endoglin/CD105 (32%)	-	-	CapG (26%)	-
	-	-	Carbonic Anhydrase IX (36%)	-	-	-	Progranulin (17%)	-
	-	-	MMP-3 (35%)	-	-	-	Serpin E1 (17%)	-
	-	-	VCAM-/CD106 (32%)	-	-	-	-	-
	-	-	HIF-1 α	-	-	-	-	-
	-	-	GM-CSF (29%)	-	-	-	-	-
	-	-	CA125 (29%)	-	-	-	-	-
	-	-	Amphiregulin (27%)	-	-	-	-	-
	-	-	VEGF (21%)	-	-	-	-	-
	-		Snail (16%)	-	-	-	-	-
	-	-	Mesothelin (16%)	-	-	-	-	-

VCAM-1, Vascular Cell Adhesion Molecule-1; CCL-2, Chemokine Ligand-2; eNOS, endothelial Nitric Oxide Synthase; FOXO1, Forkhead Box Protein O1; HCG, Human Chorionic Gonadotropin; SPARC, Secreted Protein Acidic and Rich in Cysteine; MMP-3, Matrix Metalloproteinase-3; ICAM-1, Intercellular Adhesion Molecule-1; HIF-1α, Hypoxia-Inducible Factor-1; GM-CSF, Granulocyte Macrophage-Colony Stimulating Factor; CA-125, Cancer Antigen-125; VEGF, Vascular Endothelial Growth Factor; HO-1, Hemoxygenase-1; IL-2α, Interleukin-2α; PECAM-1, Platelet Endothelial Cell Adhesion Molecule-1; CapG, Macrophage Capping Protein.

**Table 4:** Effects of curcumin treatment on invasive capacity of native and double-resistant non-small cell lung cancer (NSCLC) cell lines when in organotypic co-culture with MRC5 fibroblasts. Co-cultures were performed in triplicate on three separate occasions.

	Mean Percent Reduction in Invaded Area (±SD)					
Curcumin Concentration (μM)	A549	A549^cisR/pemR^	PC9	PC9^cisR/pemR^	PC9ER	PC9ER^cisR/pemR^
**0**	0	0	0	0	0	0
**0.25**	2.13 (±3.25)	0.43 (±3.80)	0.62 (±6.01)	19.62 (±3.17)	0.30 (±1.51)	36.66 (±1.29)
**0.5**	5.71 (±3.02)	2.88 (±3.93)	6.05 (±2.27)	21.14 (±1.97)	7.82 (±5.33)	12.92 (±0.45)
**1.0**	3.05 (±0.8)	18.87 (±1.86)	6.00 (±0.74)	35.73 (±1.68)	2.95 (±11.08)	20.13 (±1.89)
**2.5**	5.31 (±3.06)	16.10 (±1.76)	4.46 (±4.10)	34.02 (±4.03)	5.26 (±4.20)	31.41 (±1.12)
**5.0**	6.73 (±0.61)	20.33 (±2.45)	9.83 (±7.10)	53.02 (±0.55)	8.35 (±5.52)	56.59 (±0.92)

**Table 5:** Effects of HGF knockdown in MRC5 cells on invasiveness of lung cancer cell lines in organotypic co-culture. The mean percent reduction in invaded area represents change to invasion compared to their native cell line counterparts. Co-cultures were performed on three separate occasions. MRC5^-HGF^ denotes cells with stable HGF knockdown.

Cell Line Combination	Mean Percent Reduction in Invaded Area (±SD)
A549/MRC5^-HGF^	19.15% (±15.79)
PC9/MRC5^-HGF^	53.27% (±13.45)
PC9ER/MRC5^-HGF^	58.03% (±20.62)

## Supplementary Materials

The following are available online. Figure S1A: Growth curves showing A549, PC9 and PC9ER cell line response to cisplatin and pemetrexed after long-term 0.25 µM curcumin treatment. Figure S1B: Growth curves showing A549, PC9 and PC9ER cell line response to cisplatin and pemetrexed after long-term 0.25 µM curcumin treatment followed by a subsequent 3 month withdrawal period. Figure S2: Growth curves for native and doublet-resistant (R) cells in response to curcumin. Figure S3: Oncology array waterfall plot representing fold change for A549 treated with long-term, low-dose curcumin compared with native A549 cells. Figure S4: Oncology array waterfall plot representing fold change for PC9 treated with long-term, low-dose curcumin compared with native A549 cells. Figure S5: Oncology array waterfall plot representing fold change for PC9ER treated with long-term, low-dose curcumin compared with native A549 cells. Figure S6: Oncology array waterfall plot representing fold change for A549 cells treated with long-term, low-dose curcumin followed by three months of treatment withdrawal, compared with native A549 cells. Figure S7: Oncology array waterfall plot representing fold change for PC9 cells treated with long-term, low-dose curcumin followed by three months of treatment withdrawal, compared with native A549 cells. Figure S8: Oncology array waterfall plot representing fold change for PC9ER cells treated with long-term, low-dose curcumin followed by three months of treatment withdrawal, compared with native A549 cells. Figure S9: Growth curve showing effect of curcumin on MRC5 fibroblast cell number. Figure S10: Table shows HGF production in MRC5 cells following stable HGF knock-down. Figure S11: Schema showing potential ways in which curcumin may interfere with the HGF/Met signalling cascade. Figure S12: Supplemental Information for method section ‘Generation of stable HGF knock-down in MRC5 cells’.
